# Current status and future prospects of activated recombinant coagulation factor VIIa, NovoSeven®, in the treatment of haemophilia and rare bleeding disorders

**DOI:** 10.1007/s00277-023-05287-2

**Published:** 2023-06-30

**Authors:** Midori Shima

**Affiliations:** Thrombosis and Hemostasis Research Center, Nara Medical University, 840 Shijo‑cho, Kashihara City, Nara 634-8522 Japan

**Keywords:** Coagulation factor VII, Factor VII deficiency, Glanzmann thrombasthenia, Haemophilia, Recombinant factor VIIa

## Abstract

rFVIIa, a human recombinant activated coagulation factor VII, has been used worldwide for more than two decades for the treatment of bleeding episodes and prevention of bleeding in patients undergoing surgery/invasive procedures with congenital haemophilia A or B with inhibitors (CHwI A or B), acquired haemophilia (AH), congenital factor VII deficiency and Glanzmann thrombasthenia (GT), refractory to platelet transfusion. The approved dosage, administration and indication of rFVIIa in the US, Europe and Japan differ, depending on the needs of the patient population and regulatory practices. This review presents an overview of the current status and future prospects, including that from a Japanese perspective, of using rFVIIa in the treatment of approved indications. The efficacy and safety of rFVIIa in the approved indications has been demonstrated in several randomised and observational studies and data from registries. The overall incidence of thrombosis across all approved indications in a retrospective safety assessment of clinical trials and registries, prelicensure studies and postmarketing surveillance studies of rFVIIa use was 0.17%. Specifically, the risk of thrombotic events was 0.11% for CHwI, 1.77% for AH, 0.82% for congenital factor VII deficiency and 0.19% for GT. Emerging non-factor therapies such as emicizumab have changed the treatment landscape of haemophilia A, including preventing bleeding in patients with CHwI. However, rFVIIa will continue to play a significant role in the treatment of such patients, particularly during breakthrough bleeding or surgical procedures.

## Introduction

Haemophilia is a congenital disorder caused by the deficiency of factor VIII or factor IX and results in non-traumatic bleeding or prolonged bleeding after trauma or surgery. It is treated by clotting factor replacement, which often leads to the development of inhibitors and, consequently, to ineffective control of bleeding. Patients who develop inhibitors require treatment with bypassing agents (BPAs) such as recombinant activated factor VII (rFVIIa; eptacog alfa, NovoSeven® RT [1]; Novo Nordisk, Bagsvaerd, Denmark; rVIIa-jncw [eptacog beta: SEVENFACT® [2] and CEVENFACTA® [3]], approved in Europe; Laboratoire Français du Fractionnement et des Biotechnologies, S.A. [LFB S.A.], France). Among these, rFVIIa (eptacog alfa) is a human recombinant activated coagulation factor VIIa indicated for the treatment of bleeding episodes and/or prevention of bleeding during surgical/invasive procedures in congenital haemophilia A or B with inhibitors (CHwI A or B), acquired haemophilia (AH), congenital factor VII deficiency and Glanzmann thrombasthenia (GT) that is refractory to platelet transfusions or in case of unavailability of platelets for transfusion [1, 4]. Recently, FVIIa/eptacog alpha was approved by the European Medicines Agency (but not the Food and Drug Administration [FDA]) in May 2022 for the treatment of severe postpartum haemorrhage, if uterotonics are insufficient to achieve haemostasis, as it reduced the need for specific invasive procedures (one in three patients), with a very low incidence of non-fatal venous thrombotic events (one in 20 patients) [4, 5]. The approved dosage, administration and indication of rFVIIa in EU, US and Japan differ [1, 4, 6] depending on the needs of the patient population and regulatory practices (Table 1).

**Table 1:** Indications, doses and approval year for NovoSeven in the European Union, United States and Japan

	European Union [4]	United States [1]	Japan [6]
First approval	1996	1999	2000
Indication (bleeding episodes)			
Congenital haemophilia A or B with inhibitors	Approved dose [7] 1996: dose of 90 μg/kg 2007: dose of 270 μg/kg Mild-to-moderate bleeding episodes (including home therapy) • 2–3 injections of 90 μg/kg at 3-h intervals; 1 additional dose of 90 μg/kg can also be administered • 1 single injection of 270 μg/kg • Home therapy should not exceed 24 h unless provided in consultation with the haemophilia treatment centre • No data on the outcome of a single dose of 270 μg/kg in elderly patients Serious bleeding episodes • Initial dose of 90 μg/kg, which could be administered on the way to the hospital Dose adjusted based on type and severity of bleed • Initially every 2 h until clinical improvement • Every 3 h for 1–2 days if continued treatment is required • Thereafter, increase successively to 4-, 6-, 8- or 12-h intervals for as long as treatment is indicated. Major bleeds make take more than 2–3 weeks of treatment	Approved dose [7] 1999: dose of 90 μg/kg; however, a dose of 270 μg/kg is administered in clinical settings if required •90 μg/kg every 2 h, adjustable based on severity of bleeding until haemostasis • 90 μg/kg every 3–6 h after haemostasis for severe bleeding	Approved dose 2000: dose of 90 μg/kg 2013: 270 μg/kg single dose • Initial dose is 90 μg/kg (4.5 KIU/kg) every 2–3 h until haemostasis and clinical improvement, adjusted to 60–120 μg/kg (3–6 KIU/kg) based on type and severity of bleeding. For further continuation, the interval of administration is extended as appropriate • Single dose of 270 μg/kg (13.5 KIU/kg) for mild-to-moderate bleeding
Acquired haemophilia	• Initial dose of 90 μg/kg every 2–3 h; duration and interval treatment can vary with the severity of bleed • After haemostasis, increase successively to 4-, 6-, 8- or 12-h intervals for as long as treatment is indicated	• 70–90 μg/kg every 2–3 h until haemostasis	Approved in 2004 [8] for on-demand use only [9] • Initial dose is 90 μg/kg (4.5 KIU/kg) every 2–3 h until haemostasis and clinical improvement; adjusted to 60–120 μg/kg (3–6 KIU/kg) based on type and severity of bleeding. Duration and interval for further treatment extended as appropriate
Congenital factor VII deficiency	Approved in early 2000 [10] • Adults and children: 15–30 μg/kg every 4–6 h until haemostasis • Dose and frequency adapted to each individual	rFVIIa was approved for use in patients with congenital factor VII deficiency in the early 2000s in the United States [11] • 15–30 μg/kg every 4–6 h until haemostasis is achieved	Approved in 2010 for bleeding episodes • 15–30 μg/kg (0.75–1.5 KIU/kg) every 4–6 h until haemostasis; dose and frequency adjusted based on type and severity of bleeding
Glanzmann thrombasthenia	Approved in 2004 for the treatment of bleeding in patients with past or present refractoriness to platelet transfusions, or where platelets are not readily available • 90 μg (range: 80–120 μg/kg every 2 h [1.5–2.5 h]) • At least 3 doses for effective haemostasis • Recommended route of administration is bolus injection, as lack of efficacy may appear with continuous infusion • For patients who are not refractory, platelet administration is the first-line treatment	Approved in 2014 for the treatment of bleeding in patients refractory to platelet transfusion, with or without antiplatelet antibodies • 90 μg/kg every 2–6 h until haemostasis	Approved in 2011 for the treatment of bleeding in patients refractory to platelet transfusion, with or without antiplatelet antibodies • 80–120 μg/kg (4.0–6.0 KIU/kg) every 1.5–2.5 h until haemostasis and clinical improvement
Indication (perioperative management)			
Congenital haemophilia A or B with inhibitors	• Initial dose of 90 μg/kg given immediately before the intervention; repeated after 2 h and then at 2- to 3-h intervals for the first 24–48 h depending on the intervention performed and the clinical status • In major surgery, dosage continued at 2- to 4-h intervals for 6–7 days; the interval may be increased to 6–8 h for another 2 weeks of treatment • Major surgery may require 2–3 weeks of treatment until healing has occurred	Minor: • 90 μg/kg immediately before surgery, repeat every 2 h during surgery • 90 μg/kg every 2 h after surgery for 48 h, then every 2–6 h until healing has occurred Major: • 90 μg/kg immediately before surgery, repeat every 2 h during surgery • 90 μg/kg every 2 h after surgery for 5 days, then every 4 h or by continuous infusion at 50 μg/kg/hr until healing has occurred	The efficacy of this product in surgery has not been established in Japan. Perioperative indications can be based on the bleeding indications at physician’s discretion
Acquired haemophilia	1996: approved for the prevention of bleeding during surgical procedures [12] • Same as above	Approved in 2006 for the prevention of bleeding during surgical procedures [13] • 70–90 μg/kg immediately before surgery and every 2–3 h for the duration of surgery and until haemostasis	Perioperative indications can be based on the bleeding indications at physician’s discretion
Congenital factor VII deficiency	• Same as above	• 15–30 μg/kg immediately before surgery and every 4–6 h for the duration of surgery and until haemostasis	Perioperative indications can be based on the bleeding indications at physician’s discretion
Glanzmann thrombasthenia	Approved in 2004 for the prevention of bleeding during surgeries or invasive procedures in patients with past or present refractoriness to platelet transfusions, or where platelets are not readily available • Same as above	Approved in 2014 for the prevention of bleeding during surgeries or invasive procedures in patients refractory to platelet transfusion, with or without antiplatelet antibodies [1] • 90 μg/kg before surgery and repeat every 2 h for the duration of the procedure • 90 μg/kg every 2–6 h to prevent postoperative bleeding	Perioperative indications can be based on physician’s discretion after confirmation of diagnosis and assessment of need

*rFVIIa*, recombinant activated factor VII

The efficacy and safety of rFVIIa in patients with CHwI, AH, congenital factor VII deficiency and GT has been demonstrated in several randomised and observational studies [12, 14-20], as well as in studies using data from registries [21-28]. In a retrospective safety assessment of clinical trials and registries, prelicensure studies and postmarketing surveillance (PMS) studies of rFVIIa across all approved indications, the overall incidence of thrombosis was 0.17%. The risk of thrombotic events (TEs) was as low as 0.11% for CHwI, 1.77% for AH, 0.82% for congenital factor VII deficiency and 0.19% for GT, demonstrating a favourable safety profile [29]. The most common risk factors for TEs in patients receiving rFVIIa treatment were age ≥ 65 years, concomitant cardiac/vascular disease and use of activated prothrombin complex concentrate (aPCC) [29].

rFVIIa has been used extensively worldwide across indications for more than two decades. This review presents an overview of the current status and future prospects, including that from a Japanese perspective, of using rFVIIa for the treatment of approved indications.

## Methods

We performed a PubMed search from 2000 to 2021 using the search strings presented in Table 2. The aim of the search was to identify pivotal trials that define the current status and future prospects of NovoSeven® against the background of other available recombinant FVIIa and non-factor therapies. A total of 60 articles were identified for inclusion in this narrative review.

**Table 2:** Search strings

PubMed search strings (limited to year 2000 onwards)	Number of hits
("Acquired hemophilia" OR "Acquired haemophilia") AND (Novoseven OR "recombinant FVIIa" OR rFVIIa OR "activated recombinant factor VII" OR "recombinant Factor VIIa" OR "eptacog alpha" OR "eptacog alfa") AND (2000:2021[pdat])	216
("Congenital hemophilia with inhibitors" OR "Congenital haemophilia with inhibitors" OR "Hemophilia with inhibitors" OR "Haemophilia with inhibitors") AND (Novoseven OR "recombinant FVIIa" OR rFVIIa OR "activated recombinant factor VII" OR "recombinant Factor VIIa" OR "eptacog alpha" OR "eptacog alfa") AND (2000:2021[pdat])	85
("Glanzmann thrombasthenia" OR "Glanzmann's Thrombasthenia") AND (Novoseven OR "recombinant FVIIa" OR rFVIIa OR "activated recombinant factor VII" OR "recombinant Factor VIIa" OR "eptacog alpha" OR "eptacog alfa") AND (2000:2021[pdat])	129
("Congenital factor VII deficiency") AND (Novoseven OR "recombinant FVIIa" OR rFVIIa OR "activated recombinant factor VII" OR "recombinant Factor VIIa" OR "eptacog alpha" OR "eptacog alfa") AND (2000:2021[pdat])	58
("Hemophilia" OR "Haemophilia") AND (non-factor OR "non-factor agent" OR "non-factor replacement therapy" OR "emicizumab") AND (2000:2021[pdat])	319
Total	807

As eptacog beta was recently approved [2] for the treatment and control of bleeding episodes occurring in adults and adolescents (12 years of age and older) with haemophilia A or B with inhibitors, the above search strings could not be applied and the search term ‘eptacog beta’ was used on PubMed to retrieve pivotal studies on the same.

### Impact of rFVIIa on the treatment of congenital haemophilia A and B with inhibitors

Approximately 20%–30% of patients with congenital haemophilia type A and 3%–10% of those with type B develop inhibitors of factor VIII and factor IX, respectively, rendering haemostatic replacement therapies ineffective due to neutralisation [30, 31]. Inhibitors may be transient or may successfully resolve with immune tolerance induction (ITI) therapy in 60%–80% of patients with CHwI A [14]. In patients with CHwI B, the success rate of ITI is lower, particularly in those with allergy to factor IX [32]. Approximately one-third of patients with CHwI A and more than half of those with CHwI B are refractory to ITI therapy [9].

The World Federation of Hemophilia (WFH) guidelines recommend BPAs, either rFVIIa or aPCC, in patients with high-responding inhibitors (≥ 5.0 Bethesda units [BU]) with acute bleeding; they are also recommended in low-responding inhibitors, if the haemostatic response is inadequate). rFVIIa is the preferred BPA in patients with haemophilia B with high-responding inhibitors or those with severe allergy to factor IX [32].

Randomised studies conducted in patients with CHwI have reported efficacy rates for rFVIIa (eptacog alpha) of 84%–93% within 12 h [33-37], 86%–88% within 24 h [35-37]and 76%–97% within 48 h [35-37] with doses of ≤ 120 μg/kg [20]. Higher doses of ≥ 250 µg/kg have demonstrated efficacy rates of 80.5%–90.5% [34, 36], 83.3% [36] and 86.1% [36] within 12, 24 and 48 h, respectively, in the treatment of patients with CHwI and haemarthroses in home settings. Patient-reported effective resolution rates at 9 h were 63%, 60% and 56% with initial doses of ≤ 120 μg/kg, 120–250 μg/kg and ≥ 250 μg/kg among 494 spontaneous bleeding episodes documented in the ONE registry [38].

Early initiation (mean: 1.2 h) of rFVIIa after onset of bleeding has shown definite clinical improvement in pain and swelling and symptoms of nerve palsy and ischaemia along with muscle softening in 92% of peripheral intramuscular haemorrhages within 14 h, and a need for fewer doses (mean: 2.3 doses) in 116 bleeding episodes in a multicentre open-label home-treatment study in the US [30, 39]. In a real-life retrospective study conducted using data from the HemoRec registry, patients who were treated early (within 2 h of onset of bleeding) demonstrated a low rebleeding rate (4.0%–5.9%) regardless of the initial dose (< 120 to > 250 µg/kg) [26, 30], thus reinforcing the importance of early management of bleeding episodes with rFVIIa. Also, a single dose vs multiple doses of rFVIIa resulted in a lower rebleeding rate (6.3% vs 12.2%) [26]. Typically, the efficacy of a single dose (75–85 IU/kg) of aPCC is reported as comparable to two doses (90–270 µg/kg) of rFVIIa [32, 35]. Of note, neither superiority nor equivalence was demonstrated in the FENOC study, which could be attributed to the intra- and interindividual variation in response to each BPA and lack of sufficient power [35, 40]. Therefore, BPA may need to be individualised, alternated or switched according to the response, as deemed necessary [32].

In a review of studies and registries investigating rFVIIa use in home settings, on-demand treatment at standard (90 μg/kg) and high single (270 μg/kg) doses demonstrated effective haemostasis rates ranging between 81 and 96%, with very few TEs (three events: visual field defect with suspected cerebral infarction, central venous occlusion and thrombus of the arteriovenous fistula) being reported, whereas prophylactic treatment in the US demonstrated a 45%–59% reduction in bleeding frequency with no TEs [41]. Since the studies are heterogenous with respect to the definition of efficacy, patient characteristics and dosage, results should be interpreted with caution.

A retrospective analysis of the Haemophilia and Thrombosis Research Society (HTRS) registry showed that treatment with rFVIIa led to a consistently high bleeding control rate of approximately 90% across bleeding types (spontaneous, 89%; traumatic, 93%), procedure types (surgical/dental/others, 87%–93%) and bleeding locations (joint bleeding, 90%; muscle/mucosal/subcutaneous bleeding, 89%) [27]. Most bleeding episodes were controlled by a median range of 3–4 injections (dose range, 110–130 μg/kg); however, treatment of spontaneous vs traumatic bleeding with rFVIIa required higher doses per episode (median: 540 μg/kg vs 300 μg/kg), higher number of injections (median [range]: 4.0 [1–248] vs 2.5 [1–480]) and longer treatment duration (2 days vs 1 day) for spontaneous bleedings [27]. Additionally, treatment of muscle vs joint bleeding with rFVIIa required slightly higher doses per episode (median: 557 μg/kg vs 480 μg/kg) and higher number of injections (median: 4.0 vs 3.0) [27] for muscle bleedings.

A recent systematic literature review reported that varying doses of rFVIIa were effective in achieving and maintaining haemostasis, with the occurrence of very few non-haemorrhagic adverse events (AEs) in patients with CHwI undergoing non-orthopaedic or dental surgeries; one patient with several risk factors and comorbidities had myocardial infarction [16]. An analysis of two randomised trials, Hemophilia Research Society/HTRS registry databases and medical literature showed an overall effectiveness rate of 84% for rFVIIa treatment across 395 procedures (surgical, 261; dental, 89; other medical procedures, 45), with a low incidence of TEs among 263 patients with CHwI (0.4%) and 395 procedures conducted (0. 25%) [19]. Additionally, in a 10-year PMS study of rFVIIa in Japan, postoperative bleeding stopped completely or reduced considerably in 34 of 38 procedures (89%) in patients with CHwI A and in 10 of 13 procedures (77%) in patients with CHwI B [18]. Mild superficial thrombophlebitis (related to continuous infusion of rFVIIa) and mildly decreased platelet count (causal relationship with rFVIIa could not be ruled out) were reported in one patient with CHwI A each [18]. Thus, timely perioperative management with rFVIIa at approved doses reportedly results in rapid, sustained haemostasis in patients with CHwI, and is well tolerated.

In comparison, the newer rFVIIa/eptacog beta (SEVENFACT®) was shown to be pharmacodynamically active and well tolerated in a dose escalation study (25, 75 and 225 μg/kg) in non-bleeding patients with congenital haemophilia A or B [42]. Thereafter, the approval of eptacog beta (SEVENFACT® and CEVENFACTA®) was based on the outcome of PERSEPT 1–3 (randomised, phase 3 studies), which used lower doses of eptacog beta than eptacog alpha [43-45]. The PERSEPT 1 (open-label crossover trial) evaluated two initial dose regimen groups (75 µg/kg and 225 µg/kg) for 468 bleeding episodes in 27 adolescent and adult patients with haemophilia A and B with inhibitors. The reported efficacy rate was 88.7% at 12 h for both doses: 93.2% for the 225 μg/kg dose and 84.9% for the 75 μg/kg dose. The number of bleeding episodes (i.e., treatment failures) at 12 h was twice as high in the 75 μg/kg group (14.3%) vs 225 μg/kg group (6.6%). No serious AEs or deaths were reported [43].

PERSEPT 2, with a crossover design, evaluated the efficacy and safety of eptacog beta in 25 children < 12 years of age with haemophilia A or B with inhibitors. Patients received initial doses of 75 or 225 μg/kg eptacog beta, followed by 75 μg/kg dosing, based on the clinical response for mild-to-moderate bleeding episodes. The rates of treatment success at 12 and 24 h were, respectively, 65.4% and 97.4% for 239 bleeding episodes in the 75 μg/kg group vs 60.3% and 98.0% for 307 bleeding episodes in the 225 μg/kg group. Overall rates of treatment success for both doses were 62.5% at 12 h and 97.8% at 24 h. A median of two doses vs three doses was required to control bleeding in the 225 μg/kg group vs 75 μg/kg group. No TEs, inhibitors or treatment-related AEs were reported [44].

PERSEPT 3, evaluated the efficacy and safety of eptacog beta in preventing excessive bleeding in major (200 μg/kg dose) and minor (75 μg/kg dose) surgical procedures. The rate of success in six patients who underwent minor procedures was 100% and that in six patients who underwent major procedures was 66.7%; overall, 81.8% of the procedures involving eptacog beta (for primary efficacy endpoint) were considered successful. One death occurred due to bleeding, which was not attributed to eptacog beta; no TEs or anti–eptacog beta inhibitors were reported in this study [45]. A pooled safety analysis from all three phase 3 PERSEPT studies showed an excellent safety profile of eptacog beta in patients with haemophilia A or B with inhibitors for the treatment of bleeding and perioperative use [46]. A comparison between NovoSeven® and SevenFact® is presented in Table 3.

**Table 3:** Comparison of eptacog alpha (NovoSeven) with eptacog beta (SevenFact)

	NovoSeven® (Eptacog alpha)	SEVENFACT® and CEVENFACTA® (Eptacog beta)
Indication	Treatment of bleeding episodes and/or prevention of bleeding during surgical/invasive procedures in adults and children with • CHwI A or B, • Acquired haemophilia, • Congenital factor VII deficiency • Glanzmann thrombasthenia that is refractory to platelet transfusions, with or without antibodies to platelets or in case of unavailability of platelets for transfusion	rVIIa-jncw (eptacog beta; SEVENFACT®**):** Treatment and control of bleeding episodes occurring in adults and adolescents (≥ 12 years) with haemophilia A or B with inhibitors [2] rVIIa/eptacog beta **(**CEVENFACTA®**)** European Union [3]: Treatment of bleeding episodes and prevention of bleeding during surgery or invasive procedures in adults and adolescents (≥ 12 years) with • Congenital haemophilia with high-responding inhibitors to coagulation factors VIII or IX (i.e. ≥ 5 BU) • Congenital haemophilia with low-titre inhibitors (BU < 5) but expected to have a high anamnestic response to factor VIII or factor IX administration or expected to be refractory to increased dosing of FVIII or FIX
Characteristics	• rFVIIa • Produced in baby hamster kidney cells by recombinant DNA technology [4]	• rFVIIa exhibits structural (N and O glycosylation), PD and PK characteristics similar to those of activated eptacog alfa • Produced from the expression of the human factor VII gene in the mammary glands of transgenic rabbits; isolated and purified from the milk of transgenic rabbits [47, 48]
Postulated MOA	• FVII/FVIIa forms a complex with TF and activates factor IX to factor IXa and factor X to factor Xa, leading to the initial conversion of prothrombin into thrombin. Thrombin activates platelets and factors V and VIII at the site of injury and helps in the formation of the haemostatic plug by converting fibrinogen into fibrin • At pharmacological doses, it activates factor X directly on the surface of activated platelets, promoting the conversion of prothrombin into large amounts of thrombin independent of TF [4]	• FVII/FVIIa forms a complex with TF to activate FX to FXa directly, bypassing the FIX or FVIII steps in the coagulation cascade. Activation of factor X to Xa initiates the common pathway of the coagulation cascade in which prothrombin is activated to thrombin [47, 48]
Pivotal efficacy studies		
Congenital haemophilia A and B	Randomised trials Efficacy rates at a dose of ≤ 120 μg/kg [20] • 12 h: 84%–93% [33-37] • 24 h: 86%–88% [35-37] • 48 h: 76%–97% [35-37] Efficacy rate with higher doses of ≥ 250 µg/kg for hemarthroses in home settings • 12 h: 80.5%–90.5% [34, 36], • 24 h: 83.3% [36] • 48 h: 86.1% [36] Registries Patient-reported effective resolution rates at 9 h for 494 spontaneous bleeding episodes [38] • ≤ 120 μg/kg: 63% • 120–250 μg/kg: 60% • ≥ 250 μg/kg: 56% HemoRec registry (retrospective study) [26] Patients treated early (within 2 h of bleeding) with initial dose (< 120 to > 250 µg/kg) [26, 30], • Rebleeding rate: 4.0%–5.9% • Rebleeding rate (single dose vs multiple doses: 6.3% vs 12.2%) HTRS registry (retrospective study) [27] • Bleeding control rate: ⁓90% (required median range of 3–4 injections [dose range, 110–130 μg/kg]) • Spontaneous: 89% (median injection, median dose, duration: 4.0, 540 μg/kg, 2 days) • Traumatic: 93% (median injection, median dose, duration: 2.5, 300 μg/kg, 1 day) • Surgical/dental/other procedure: 87%–93% • Muscle/mucosal/subcutaneous bleeding: 89% (median dose, median injections: 557 μg/kg, 4.0) • Joint bleeding: 90% (median dose, median injections: 480 μg/kg, 3.0) Two RCTs/HRS/HTRS registry/medical literature [19] • Overall effectiveness rate: 84% (395 procedures [surgical, 261; dental, 89; other medical procedures, 45]), • TE rates: 0.4% of 263 patients and 0.25% of 395 procedures 10-year PMS study (Japan), postoperative bleeding control [18] • CHwI A: 89% (34 of 38 procedures) • CHwI B: 77% (10 of 13 procedures) • AEs: Mild superficial thrombophlebitis and mildly decreased platelet count (causal relationship with rFVIIa could not be ruled out) were reported in 1 patient with CHwI A each	Randomised trials PERSEPT 1 [43] Efficacy rates (mild-to-moderate BEs) • 12 h: 88.7% overall success proportion • 12-h traumatic BEs: 90.1% • 12-h spontaneous BEs: 88.5% 75 μg/kg dose • 12 h: 84.9% • 24 h: 96.3% 225 μg/kg dose • 12 h: 93.2% • 24 h: 96.3% Additional 75 μg/kg dose (treatment failure) • 12 h: 14.3% of BEs • 24 h: 3.1% of BEs required alternative therapy Additional 225 μg/kg (treatment failure) • 12 h: 6.6% of BEs • 24 h: 0.47% of BEs required alternative therapy No SAEs and deaths PERSEPT 2 [44] Efficacy rates (mild-to-moderate BEs, success proportion) Overall • 12 h: 62.5% • 24 h: 97.8% 75 μg/kg dose • 12 h: 65.4% • 24 h: 97.4% 225 μg/kg dose • 12 h: 60.3% • 24 h: 98.0% No TEs, allergic reactions, inhibitors or TRAEs PERSEPT 3 (Not an RCT)[45] Success rate (*n* = 12) Intraoperative efficacy evaluation • Minor procedure: 100% • Major procedure: 100% Efficacy, 24 h ± 2 after procedure completion • Minor procedure: 100% • Major procedure: 66.7% Primary efficacy endpoint (48 h ± 4 after last dose) • Minor procedure: 100% • Major procedure: 66.7% AEs: 3 AEs possibly/probably related in 1 patient who was discontinued (2 were SAEs) Pooled safety of all studies [46]**:** • No TEs • TRAEs: 10 in 3 patients • Immunogenicity: 1 patient

*AE*, adverse event; *BE*, bleeding episode; *BU*, Bethesda unit; *CHwI *A or B, congenital haemophilia A or B with inhibitors;* EPCR*, Endothelial cell protein C receptor; *FDA*, Food and Drug Administration; *HRS*, Haemophilia Research Society; *HTRS*, Haemophilia and Thrombosis Research Society; *PD*, pharmacodynamic; *PK*, pharmacokinetic; *RCT*, randomised clinical trial; *rFVIIa*, recombinant activated factor VII; *SAE*, serious adverse event; *TE*, thrombotic event; *TF*, tissue factor; *TRAE*, treatment-related adverse event

### Impact of rFVIIa on the treatment of AH

AH is rare condition and occurs due to the development of inhibitors against coagulation factor VIII. The incidence of AH is estimated to be 1.5 per million per year [49]. AH may present as no bleeding or bleeding episodes, which range in severity from mild to life-threatening. A provisional diagnosis of AH is considered in a patient with an acute or recent onset of bleeding and unexplained prolonged activated partial thromboplastin time (aPTT) and further confirmed by the Bethesda assay and/or an anti–factor VIII enzyme-linked immunosorbent assay [50].

According to the updated international AH treatment recommendations based on European and US registries, BPAs or recombinant porcine factor VIII is recommended for the initial treatment of clinically relevant bleeding in patients with AH and for prophylaxis in patients with AH who are undergoing invasive procedures [50].

rFVIIa has demonstrated efficacy and safety in patients with AH. Data from the largest North American AH patient series, HTRS and European Acquired Haemophilia (EACH2) registries showed that rFVIIa was used in more than 50% of treated cases as a BPA [17, 22, 51]. A study of the HTRS registry showed that among 139 reported bleeding episodes treated with rFVIIa (median initial dose, 90 µg/kg; first line, 127; second line, 12), bleeding stopped in 85%, reduced in 11% and showed ‘no improvement’ in 4%, per the physician’s rating. A single episode of transient TE was reported postpartum in a woman who had received 110 doses of rFVIIa [22]. A systematic literature review of 12 studies comprising 1244 patients with AH and 1714 bleeding episodes reported > 90% haemostatic effectiveness of rFVIIa (median number of doses, 10–28) in resolving bleeding or achieving a complete or partial response [12]. The safety profile of rFVIIa was generally favourable, with a low risk of AEs, including TEs.

The Acquired Hemophilia Surveillance project, a simple case report surveillance that collected safety data from healthcare professionals who were excluded from HTRS participation, reported no AEs with a possible/probable relation with rFVIIa treatment in 65 cases of AH [52]. At the same time, haemostasis outcomes in the HTRS registry were assessed as ‘excellent’, ‘good’ or ‘no other haemostatic agent required’ in 91% of the 22 surgical and other invasive procedures in patients with AH who received rFVIIa infusion (median dose, 91.5 [43–200] µg/kg per episode) before (*n* = 11)/after (*n* = 13) and during (*n* = 6) the procedures [13].

In the largest, single-country experience, a PMS study conducted from 2000 to 2010 in Japanese patients with AH demonstrated improvement in bleeding in 91% of 302 bleeding episodes among 132 patients with AH using rFVIIa monotherapy (median, three doses, 93.2 µg/kg) as a first-line treatment [15]. The response rate was significantly improved in patients who received an initial dose of ≥ 90 µg/kg (vs < 90 µg/kg) and received the first rFVIIa dose promptly following the onset of bleeding. Five serious TEs were reported in three elderly patients with significant comorbidities.

A safety review reported a TE prevalence of < 4 in 100,000 across 800,000 standard doses (90 μg/kg for a patient weighing 40 kg) of rFVIIa administered over approximately 3.5 years in patients with CHwI and AH [53]. In cases of CHwI and AH, concomitant use of rFVIIa and aPCC because of low efficacy with either of these agents when used alone increased the risk of thrombosis substantially, although efficacy improved as well. However, such off-label concomitant use should be avoided or attempted under medical supervision at the physician’s discretion (if required) and not at home. Therefore, further studies are warranted to explore concomitant administration of two BPAs [54]. On the other hand, sequential use of rFVIIa and aPCC following unsatisfactory haemostasis with single-agent treatment is recommended and well documented [32]. The WFH recommends that if haemostasis is unsatisfactory with rFVIIa or aPCC as single agents, then each may be alternated every 6 h at a dose of 90 µg/kg rFVIIa and 50 U/kg aPCC [32, 55, 56]. However, combination/sequential bypass agent treatment should be limited to treatment centres with extensive experience in managing haemophilia patients with inhibitors because close monitoring for thrombosis and disseminated intravascular coagulation is vital. Because of the presence of FVIII in aPCC, it is estimated that aPCC treatment leads to an anamnestic response in approximately 30% of patients with FVIII inhibitors [32, 57]. A small retrospective study (*n* = 4) and web-based database search (*n* = 11) reported that sequential therapy with aPCC and rFVIIa controlled bleeding in 12–24 h in patients with unresponsive bleeding due to inhibitors. Both studies did not report AEs [58, 59]. Another small retrospective study (*n* = 4) also reported resolution of bleeds after a median of 3 days of sequential therapy in patients with refractory bleeding episodes with haemophilia.and inhibitors [60].

### Use of rFVIIa in rare bleeding disorders

#### Congenital factor VII deficiency

Congenital factor VII deficiency is a rare, autosomal recessive disorder resulting from mutations in the gene encoding coagulation factor VII, with an estimated prevalence of 1 per 500,000 in the general population [61]. These patients commonly present with easy bruising, epistaxis, gum bleeding and menorrhagia or bleeding after surgery, although there is a substantial variation in clinical symptoms among patients [61]. Aggressive replacement therapy with or without long-term prophylactic treatment is considered in patients with severe symptoms [28], which comprise 10%–15% of those affected [24]. The year of approval and doses of rFVIIa for congenital factor VII deficiency are presented in Table 2.

The recommended dose of rFVIIa (15–30 µg/kg every 4–6 h) [1] for achieving haemostasis in patients with congenital factor VII deficiency is considerably lower than that for patients with haemophilia (90 µg/kg every 2–3 h) [10]. The lower dose requirement is likely a result of the intact factor XI-feedback loop in patients with congenital factor VII deficiency, which amplifies the initial thrombin generation to a thrombin burst [10].

A study conducted using data from the Seven Treatment Evaluation Registry (STER) reported cessation of spontaneous and traumatic bleeding episodes in 92% (*n* = 73) of 79 episodes with single (*n* = 38) and multiple (*n* = 35) doses (median: 60 µg/kg) of rFVIIa in patients with congenital factor VII deficiency [28]. Further, 21 of 34 patients with severe congenital factor VII deficiency at 13 HTCs across 11 countries received prophylaxis (off-label) with rFVIIa in the web-based STER [24]. No bleeding episodes, particularly in target sites, were reported in 14 of 22 courses of prophylactic rFVIIa with total weekly median doses of 90 µg/kg (1–3 doses per week) [24]. The surgical study conducted using STER data in patients with congenital factor VII deficiency concluded that the administration of ≥ 3 doses of rFVIIa (≥ 13 µg/kg/body weight per dose) on the day of surgery is required for effective haemostasis [23]. Bleeding was reported in only 3 of 41 orthopaedic surgeries, all of which were major high-risk surgeries wherein patients received a very low dose of rFVIIa. No episodes of thrombosis were reported during follow-up [23, 24].

#### Glanzmann thrombasthenia

GT is a rare, autosomal recessive bleeding syndrome characterised by a lack of platelet aggregation, resulting from a mutation in αIIbβ3 integrin, an essential component for optimal platelet function and haemostasis [62, 63]. GT is prevalent in 1 of 1,000,000 individuals; however, the prevalence is reportedly higher in ethnic groups such as Iraqi Jews, selective Arab populations (e.g. Palestinians) and French Gypsies, where consanguineous marriages are common [64].

Clinical presentation is highly variable, ranging from mild bruising to severe life-threatening haemorrhages [62]. Although bleeding episodes can be managed by local measures alone or in combination with antifibrinolytics (AF), platelet transfusions may be required to control or prevent life-threatening haemorrhages [64]. GT may be further complicated due to the development of isoantibodies against αIIbβ3 integrin in 20%–30% of patients [65], rendering them refractory to platelet transfusion [21]. Early stratification of patients with GT at high risk of antibody formation can help in individualising treatment by avoiding platelet transfusion, thereby preventing antibody formation [65]. Furthermore, early recognition and referral of such patients to experienced haematologists can improve treatment outcomes by optimising long-term management [66].

Although the exact mechanism of the haemostatic action of rFVIIa in GT is not known, it is speculated that rFVIIa helps in the formation of a stable and effective clot by enhancing platelet activation, thrombin generation and fibrin deposition [25]. Data from a prospective, postmarketing, observational, web-based registry of patients with GT from 15 countries, the Glanzmann’s Thrombasthenia Registry (GTR), showed that treatment was judged as effective, implying that bleeding stopped and haemostasis was achieved for at least 6 h in 91% of bleeding episodes treated with rFVIIa alone compared with 72.7% of bleeding episodes treated with rFVIIa in combination with AF and platelets [21]. Additionally, the rebleeding rate was reported as 0.95% with rFVIIa alone and 21.1% in combination with platelets ± AF. Patients with severe (vs moderate) bleeding with (vs without) a history of inhibitors and/or refractoriness to platelets received a higher number of rFVIIa doses and cumulative doses and had a greater treatment duration. rFVIIa alone or in combination with AF and platelets was not associated with any TEs in non-surgical bleeding [21].

According to an independent adjudication analysis of the GTR registry by a haematology expert panel in the US, rFVIIa was considered successful in achieving haemostasis in 94.4% of 266 bleeding episodes and 99.4% of 160 surgical procedures [25]. In the Novo Nordisk safety surveillance database for GT, 42 AEs, 24 of them serious, were observed and considered as identified risks associated with rFVIIa use. The AEs included TEs (*n* = 15; one case of arterial, nine cases of venous and five cases of mixed TEs) and allergic reactions (*n* = 3); lack of efficacy was observed in 24 patients [67].

### Emergence of non-factor therapies and their impact on the use of rFVIIa in haemophilia

Emicizumab, a bispecific factor IXa- and factor X-directed antibody that was the first non-factor replacement therapy approved in the US in 2017 and in the EU and Japan in 2018 [68], offered an alternative treatment approach for patients with haemophilia [32]. It is approved for routine prophylaxis to prevent or reduce the frequency of bleeding episodes in adult and paediatric patients with haemophilia A with or without inhibitors [69, 70]. Emicizumab has reduced the need for frequent intravenous injections in patients with CHwI. Moreover, episodes of breakthrough bleeding are reduced with emicizumab prophylaxis [71].

The WFH recommends emicizumab for regular prophylaxis in patients with haemophilia A with inhibitors [32]. Long-term, pooled results from the multicentre, open-label, phase 3 studies, HAVEN 1–4, have demonstrated low bleeding rates in patients with haemophilia A with or without inhibitors, with 970.3 patient-years of exposure of emicizumab and a well-tolerated safety profile [72]. Over a follow-up of 18 months, spontaneous and traumatic episodes (≥ 1) of breakthrough bleeding were reported in 51% and 61% of patients on emicizumab prophylaxis in a real-world setting, respectively [73]. Thus, emicizumab requires careful monitoring when administered in elderly patients for prophylaxis.

Emicizumab is not intended for the treatment of acute bleeding episodes or breakthrough bleeding that occurs between prophylactic doses. Consequently, breakthrough bleeding requires intervention with factor concentrates or with a BPA in patients with inhibitors [32]. However, the risk of thrombosis is potentially increased with emicizumab [71], particularly in combination with high and frequent doses (> 100 U/kg/24 h for 24 h or more) of aPCC [32, 69].

Thrombotic complications have not been reported when concomitant rFVIIa is administered; however, caution is urged in patients with risk factors for thrombosis [32, 74]. In all acute and severe bleeding episodes, including those that are life-threatening; involve critical organs, muscles or joints; or cause a drop in haemoglobin by 20 g/L or more while the patient is receiving emicizumab, rFVIIa can be administered as the first-line treatment [32, 74]. rFVIIa is recommended at doses expected to achieve haemostasis [32]: 1–3 doses of 90–120 μg/kg administered at a frequency of less than every 2 h [7].

rFVIIa may also be required as first-line adjunctive therapy in case of acute bleeding manifestations due to trauma, invasive procedures or emergency/elective surgery in CHwI patients treated with emicizumab. Additional injections of emicizumab are not recommended in the emergency setting [74]. rFVIIa is the recommended first-line treatment both pre- and postoperatively in case of non-minor surgeries at a starting dose of 90 µg/kg, which may be repeated, and the dose frequency gradually reduced by increasing dose intervals [74]. Ease of subcutaneous administration, less frequent dosing and steady-state correction achieved with emicizumab support its use in patients with CHwI. However, rFVIIa may be additionally required during certain emergencies [74]. Based on a review of evidence from 2016 to 2020 (case reports and retrospective chart review), surgery can be successfully performed with the concomitant use of rFVIIa and emicizumab; however, this needs to be confirmed using prospective studies [75]. Currently, eptacog beta is being evaluated in a phase IV, US-centric, open-label, safety study in patients with haemophilia A or B with inhibitors who are either receiving or not receiving long-term prophylactic treatment (e.g., emicizumab) and are at risk of experiencing a breakthrough bleeding event [76].

Another non-factor therapy concizumab binds to the Kunitz-2 domain of tissue factor pathway inhibitor (TFPI), thereby preventing TFPI from binding to the active site of factor Xa, making sufficient FXa available from the FVIIa-tissue factor complex to achieve haemostasis [77]. The concomitant use of rFVIIa with concizumab is in experimental phase [78, 79]. On the basis of phase 2 results, the FDA granted concizumab a breakthrough therapy designation for patients with haemophilia B with inhibitors, a rare and vulnerable patient subgroup that currently has the highest unmet medical need [77, 80, 81]. Compassionate use of concizumab in severe haemophilia A and B, where the potential benefit justifies the potential risks of treatment, is also being evaluated [82].

All other non-factor therapies including fitusiran (an antithrombin inhibitor antibody), Mim8 (next-generation factor VIIIa mimetic) and NXT007 (emicizumab-based engineered Fixa/Fx bispecific antibody with improved properties) for the treatment of both haemophilia A and B are now in the experimental phase.

## Conclusions

The need for an individualised management strategy and current availability of various treatment options for haemophilia make it pertinent for the treating physicians to understand each product profile and patient characteristics to ensure optimal management of bleeding disorders. In case of rare bleeding disorders, the small patient population makes it difficult to conduct large-scale studies to inform treatment approaches. Furthermore, in the absence of evidence-based guidelines, there is a need for achieving consensus on treatment recommendations for rare bleeding disorders through expert forums and webinars.

Registries and/or PMS studies in patients with congenital factor VII deficiency, GT and AH who receive rFVIIa treatment have enabled the collection of efficacy and safety data to understand the real-world management of these rare bleeding disorders, which may help in better defining the dose and criteria for the use of rFVIIa in this population. rFVIIa has played a vital role in the management of patients with haemophilia and certain rare bleeding disorders over two decades. It continues to be a key BPA for the treatment of breakthrough bleeding and prophylactic use prior to invasive procedures in the rapidly changing treatment landscape of emerging non-factor therapies.
